# iNKT/CD1d-antitumor immunotherapy significantly increases the efficacy of therapeutic CpG/peptide-based cancer vaccine

**DOI:** 10.1186/s40425-014-0039-8

**Published:** 2014-11-18

**Authors:** Stéphanie Corgnac, Rachel Perret, Lianjun Zhang, Jean-Pierre Mach, Pedro Romero, Alena Donda

**Affiliations:** Translational Tumor Immunology Group, Ludwig Center for Cancer Research, University of Lausanne, Lausanne, Switzerland; Department of Biochemistry, University of Lausanne, Lausanne, Switzerland

**Keywords:** iNKT cells, CD1d, CpG-based vaccine, Combined cancer immunotherapy

## Abstract

**Background:**

Therapeutic cancer vaccines aim to boost the natural immunity against transformed cancer cells, and a series of adjuvants and co-stimulatory molecules have been proposed to enhance the immune response against weak self-antigens expressed on cancer cells. For instance, a peptide/CpG-based cancer vaccine has been evaluated in several clinical trials and was shown in pre-clinical studies to favor the expansion of effector T versus Tregs cells, resulting in a potent antitumor activity, as compared to other TLR ligands. Alternatively, the adjuvant activity of CD1d-restricted invariant NKT cells (iNKT) on the innate and adaptive immunity is well demonstrated, and several CD1d glycolipid ligands are under pre-clinical and clinical evaluation. Importantly, additive or even synergistic effects have been shown upon combined CD1d/NKT agonists and TLR ligands. The aim of the present study is to combine the activation and tumor targeting of activated iNKT, NK and T cells.

**Methods:**

Activation and tumor targeting of iNKT cells via recombinant α-galactosylceramide (αGC)-loaded CD1d-anti-HER2 fusion protein (CD1d-antitumor) is combined or not with OVA peptide/CpG vaccine. Circulating and intratumoral NK and H-2Kb/OVA-specific CD8 responses are monitored, as well as the state of activation of dendritic cells (DC) with regard to activation markers and IL-12 secretion. The resulting antitumor therapy is tested against established tumor grafts of B16 melanoma cells expressing human HER2 and ovalbumin.

**Results:**

The combined CD1d/iNKT antitumor therapy and CpG/peptide-based immunization leads to optimized expansion of NK and OVA-specific CD8 T cells (CTLs), likely resulting from the maturation of highly pro-inflammatory DCs as seen by a synergistic increase in serum IL-12. The enhanced innate and adaptive immune responses result in higher tumor inhibition that correlates with increased numbers of OVA-specific CTLs at the tumor site. Antibody-mediated depletion experiments further demonstrate that in this context, CTLs rather than NK cells are essential for the enhanced tumor inhibition.

**Conclusions:**

Altogether, our study in mice demonstrates that αGC/CD1d-antitumor fusion protein greatly increases the efficacy of a therapeutic CpG-based cancer vaccine, first as an adjuvant during T cell priming and second, as a therapeutic agent to redirect immune responses to the tumor site.

**Electronic supplementary material:**

The online version of this article (doi:10.1186/s40425-014-0039-8) contains supplementary material, which is available to authorized users.

## Background

The importance of immune surveillance in eradicating malignant cells is well demonstrated, and several observations have motivated the development of therapeutic cancer vaccines. However, except for virus-induced cancers, tumor antigens are mostly self or near-self protein epitopes that are often poorly immunogenic and submitted to central and peripheral tolerance. For therapeutic cancer vaccines to be effective, they must overcome regulatory and immunosuppressive mechanisms raised by the immune system itself and by the tumor microenvironment [1,2]. It is now well demonstrated that DCs are the central cell population making the decision between immunity or immune tolerance, depending on the stimuli that they receive [2,3]. For instance, the development of tolerogenic DCs will lead to antigen tolerance in particular through the expansion of T regulatory cells (Tregs). We have recently shown that the vaccine formulation, and in particular the presence of the Toll-like Receptor (TLR) agonist CpG, can significantly promote the maturation of pro-inflammatory DCs, which favors Type I T cell responses while restricting the expansion of Tregs [4]. In addition to TLR ligands, CD1d-restricted invariant NKT (iNKT) cells have been shown to efficiently promote the transactivation of DCs through the CD40L-CD40 interaction upon recognition of the CD1d-glycolipid antigen complex by their semi-invariant TCR. In view of the capacity of iNKT cells to promote DC maturation and NK cell transactivation, several pre-clinical studies have investigated the use of the CD1d/iNKT super agonist α-galactosylceramide (αGalCer), or a related analog as vaccine adjuvant [5–7]. More recently, several studies have demonstrated a cooperative effect on DC maturation between TLR ligands and iNKT cell activation, resulting in highly pro-inflammatory DCs, as seen by enhanced expression of activation markers [8–10]. However, if generating pro-inflammatory DCs should result in a good expansion of antigen-specific T cells, it will not guarantee their efficient homing to the tumor, unless a targeting strategy is used. In this context, we have developed CD1d-antitumor fusion proteins consisting of the soluble part of the CD1d molecule fused to a scFv antibody fragment specific for the tumor antigens CEA or HER2. We have previously demonstrated *in vitro* and *in vivo* that these fusion proteins were able to redirect iNKT, NK and T cells to the tumor expressing the relevant antigen resulting in a potent antitumor effect [11,12]. In the present study, we aimed to combine a CpG-based peptide vaccine with the activation and tumor targeting of iNKT cells via the CD1d-anti-HER2 fusion protein.

## Results

### αGC/CD1d-mediated activation of iNKT cells combined with CpG-ODN promote highly pro-inflammatory DCs

**Figure 1:**
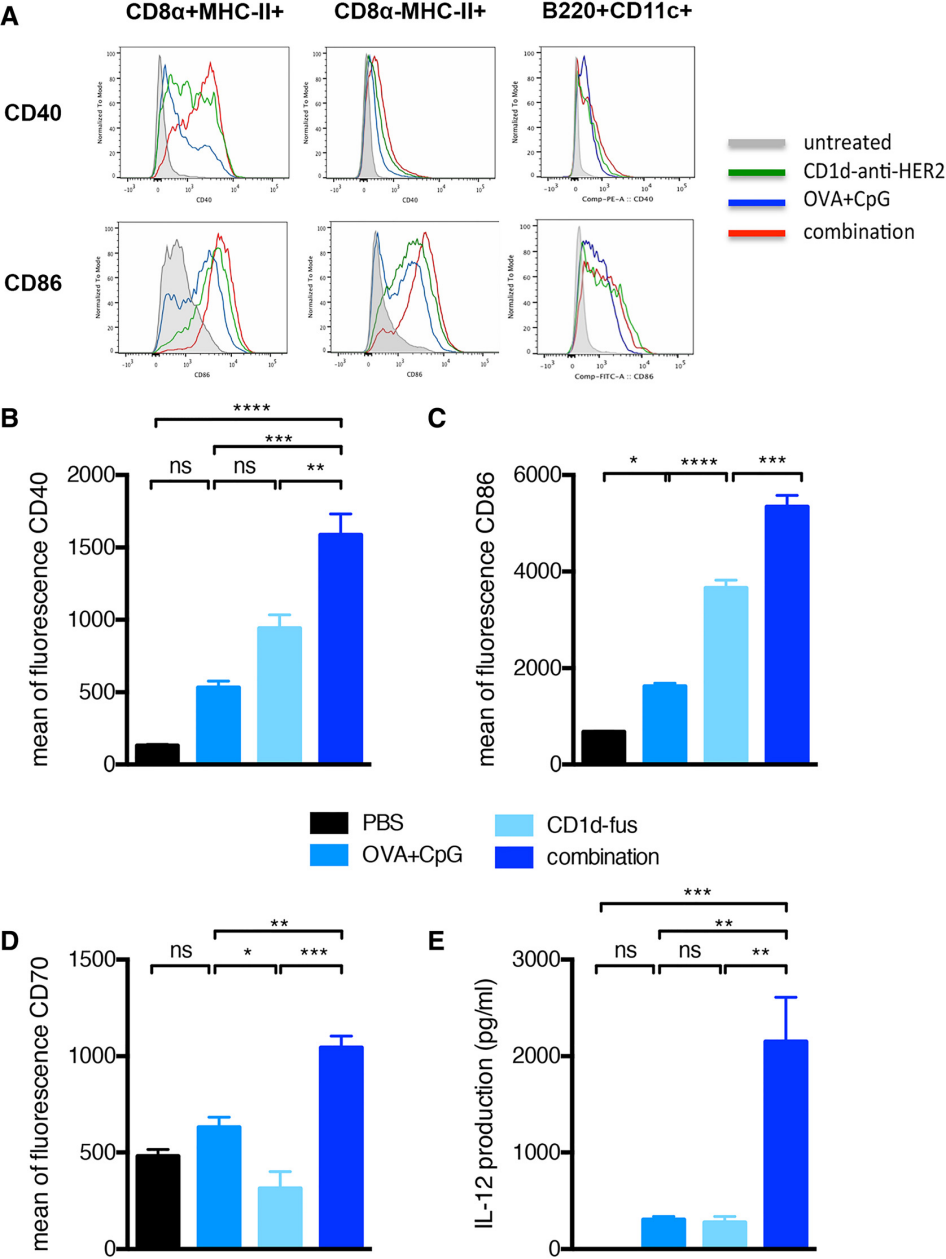
**Optimal maturation of CD8**α^**+**^ **DCs after CD1d-mediated activation of iNKT cells combined with CpG-ODN.** Mice transferred with Vα14-Jα18 and OT-I splenocytes were immunized i.m. with OVA peptide alone or in combination with CpG or CD1d-fus/CpG followed or not by systemic treatment with the CD1d-antitumor protein. Mice were bled after 6 hours and sacrificed 20 hours after i.m. immunization. **A**. Twenty hours post immunization, splenic DC subsets were discriminated according to the gating strategy described in Additional file 1: Figure S1, and each subset was analyzed for the expression of CD40 and CD86 maturation markers. Data are shown as histogram overlays of the different treatment groups as indicated. **B**. Mean fluorescence intensity of CD40 on gated CD8α^+^DCs. **C**. Mean fluorescence intensity of CD86 on gated CD8α^+^DCs. **D**. Mean fluorescence intensity of CD70 on gated CD8α^+^DCs. **E**. Sera from mice were collected 6 hours after the immunization and IL-12p70 was measured using a BD flex set assay. Bar graphs show mean of fluorescence or cytokine level in pg/ml as mean +/− SEM for 3 samples per group. Data are representative of 2 independent experiments. *, p <0.05; **, p <0.01; ***, p <0.001; ****, p <0.0001.

We, and others, have previously reported that iNKT cells and TLR ligands are both potent inducers of pro-inflammatory DCs [2,4,7]. In the present study, we evaluated the possible synergy on the maturation of DCs when combining the αGC/CD1d-antitumor fusion protein treatment with CpG-based peptide vaccine. In this regard, mice transferred with Vα14-Jα18 and OT-I splenocytes and immunized with OVA peptide alone or in combination with CpG or CD1d-fus/CpG were sacrificed 20 hours after i.m. immunization. Indeed, expression of CD40, CD86, and MHC-II on CD8α^+^ CD11c^+^ DCs, as identified by using the gating strategy depicted in Additional file 1: Figure S1, was enhanced upon treatment with the combined stimuli, as compared to the CD1d-scFv fusion or CpG as single agent (Figure 1B and C and data not shown). Moreover, the expression of CD70 essential for the priming of naïve CD8 T cells [13,14] was not affected by each stimulus alone, but was significantly up-regulated upon the combined CD1d-fusion and CpG treatment (Figure 1D). Interestingly, the up-regulation of CD86 and CD40 was much weaker in the CD8α^neg^ DC and B220^+^CD11c^+^ pDC subsets, and was similar upon individual or combined treatments (Figure 1A). These observations strongly suggest that CD8α^+^ DCs were the main DC population simultaneously triggered through TLR-9 by CpG ODN and by activated iNKT cells via CD40-CD40L. Finally, the major observation was the synergistic production of IL-12p70 in the serum six hours after the combined treatment (Figure 1E). Indeed, the circulating level of this cytokine was ten times higher in mice treated with the combination of CD1d-fusion and OVA/CpG-ODN than with the CD1d-fusion or OVA/CpG-ODN alone (Figure 1E). Altogether, these results demonstrate the potential of combined activation of iNKT cells and direct stimulation with a TLR-9 ligand in promoting highly pro-inflammatory cross-presenting CD8α^+^ DCs.

### Optimal priming of OVA-specific CTLs and expansion of NK cells upon combined CD1d treatment and OVA/CpG immunization

**Figure 2:**
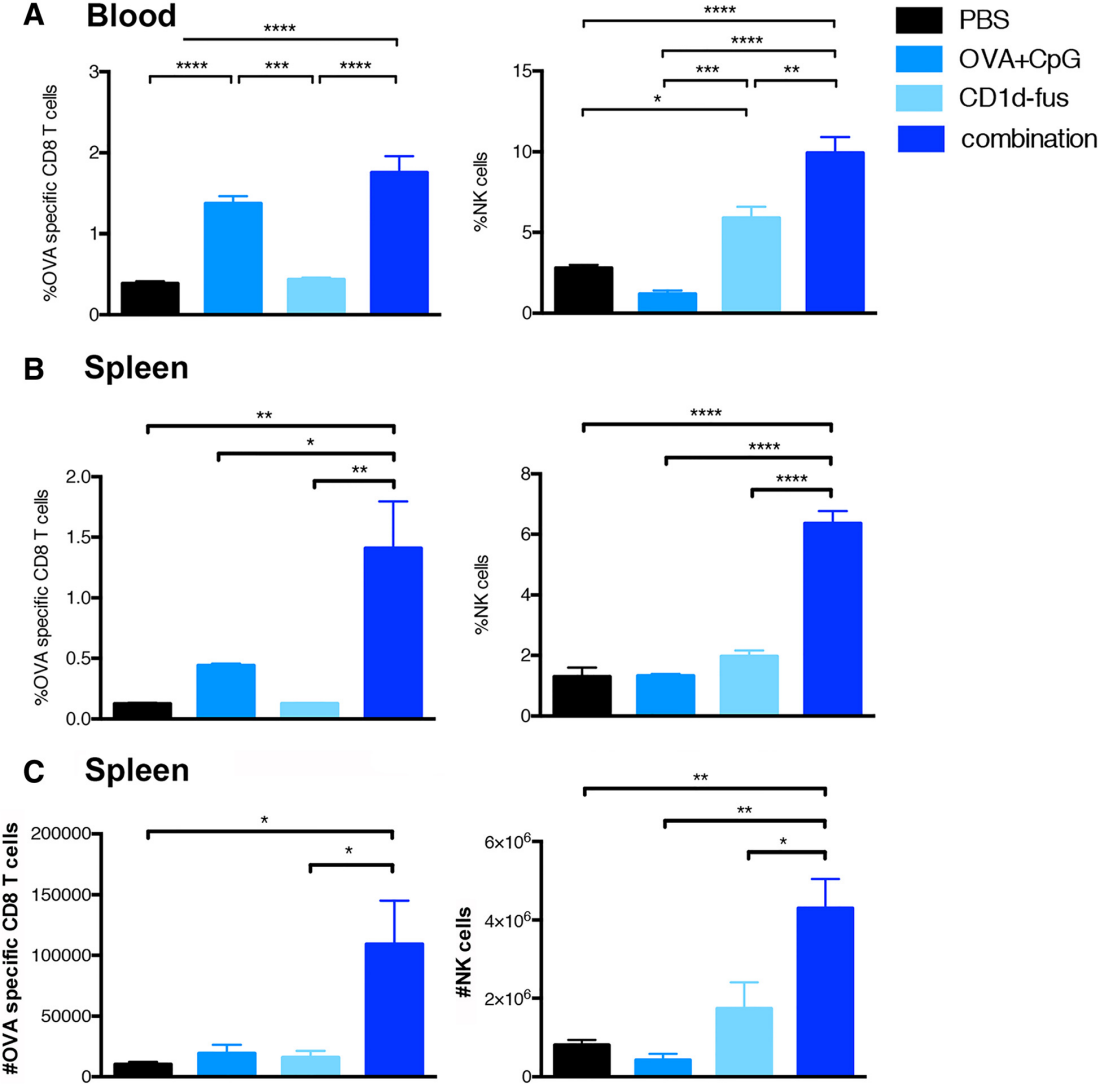
**Expansion of OVA-specific CTLs and NK cells upon combined CD1d treatment and OVA/CpG immunization.** Blood and spleen lymphocytes from CD45.1 mice that received Vα14-Jα18 and OT-I cells and were immunized and treated as in Fig. 1, were monitored at different time points after immunization. **A**. Left; frequency of H-2K^b^/OVA-specific CD8 T cells among total CD8 lymphocytes in the blood at day 3 after immunization. Right; frequency of NK cells in the blood at the same time point. **B**. Left; frequency of H-2K^b^/OVA-specific CD8 T cells among total CD8 lymphocytes in the spleen at day 7 after the immunization. Right; frequency of NK cells among total lymphocytes at the same time point. **C**. Left; total numbers of H-2K^b^/OVA-specific CD8 T cells in the spleen at day 7. Right; total numbers of NK cells in the spleen at the same time point. Bar graphs show frequencies as mean +/− SEM of groups of 5 or 3 mice. Data are representative of 3 independent experiments. *, p <0.05; **, p <0.01; ***, p <0.001; ****, p <0.0001.

Next, we evaluated whether the optimal maturation of CD8α^+^ DCs would result in increased T cell priming after intramuscular administration of the CD1d-immunotherapy together with OVA/CpG-ODN vaccine in mice transferred with Vα14-Jα18 and OT-I cells. Indeed, already at day 3 post-immunization, increased frequencies of OVA-specific CD8 T cells were observed in the blood of mice treated with the combined treatment, as compared to the OVA/CpG-ODN immunization alone (Figure 2A, left). The superior CD8 T cell priming with the combined settings reached statistical significance at day 7 in the spleen, when compared to all the other groups, including OVA/CpG-ODN immunization alone (Figure 2B, left). Activated iNKT cells are well known to transactivate NK cells [15,16], as further confirmed in the present study with the CD1d-fusion treatment (Figure 2A and B, right). Interestingly, the combined CD1d immunotherapy and peptide/CpG-ODN immunization resulted in a synergistic effect on the expansion of NK cells with a threefold increased frequency at day 7 in the spleen (Figure 2B, right), while peptide/CpG-ODN vaccine alone had no effect on NK cells. The increase in CD8 and NK cell frequencies in response to the combined stimuli was mirrored by an increase in total cell numbers (Figure 2C). Finally, an additive effect of combined stimuli on NK cell activation was already seen in the spleen twenty hours after immunization by CD69 up-regulation (Additional file 2: Figure S2A). Whereas iNKT cell-mediated transactivation of DCs, CTLs and NK cells was clearly seen by up-regulation of surface markers and cell expansion, the *in vivo* activation of iNKT cells themselves was rather revealed by their quick disappearance due to their TCR down-regulation (data not shown). However, their fast activation by the CD1d-anti-HER2 fusion protein could be detected *in vitro* by their mild increased frequency and by a significant up-regulation of CD69 (Additional file 2: Figure S2B).

In light of the robust response induced by combination therapy in the OVA model, we sought to test whether it would also work successfully as a stand-alone therapy to generate endogenous immune responses against a natural tumor/self antigen. To test this hypothesis, we vaccinated mice with the peptide Trp2_180–188_ from the melanoma antigen tyrosinase-related protein 2, either alone, with CpG-ODN, or in combination with both CpG-ODN and CD1d-HER2 fusion protein. In Additional file 3: Figure S3A, we show that the combination treatment significantly increased the frequency of endogenous H-2Kb/Trp2-specific CD8^+^ T cells over that of the peptide vaccine alone. There was also a slight increase over the peptide + CpG-ODN vaccine. Additionally, NK cell frequencies were increased, over both the peptide and peptide + CpG-ODN vaccines in response to the combination treatment, to the same extent as in the OT-I model (Additional file 3: Figure S3B). Altogether, the CD1d-mediated activation of iNKT cells combined with the TLR9 ligand resulted in an overall synergistic effect on the innate and adaptive immune responses.

### Enhanced serum levels of TH1 cytokines upon combined CD1d treatment and OVA/CpG immunization

**Figure 3:**
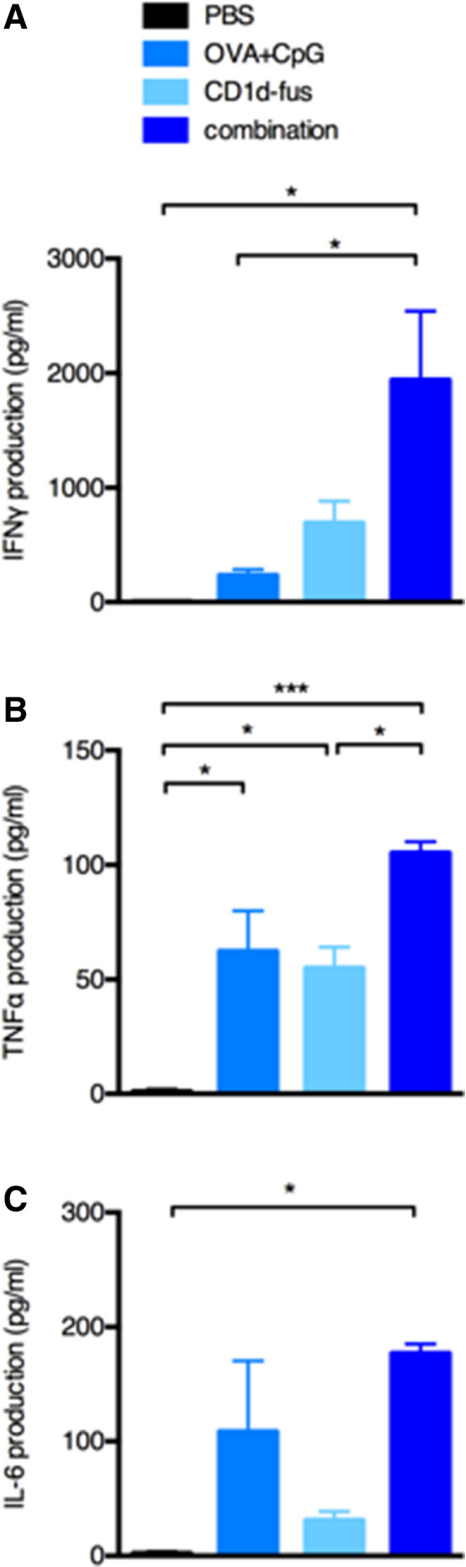
**Serum levels of cytokine upon combined CD1d treatment and OVA/CpG immunization.** Mice were immunised i.m. with OVA peptide alone or in combination with CpG or CD1d-fus/CpG following Vα14-Jα18 and OT-I cell transfer and treated or not systemically with CD1d-fus. Sera were collected 20 hours after the different immunisations and cytokine levels were measured using the Th1/Th2/Th17 CBA assay. **A**. Serum IFNγ level. **B**. Serum TNFα level. **C**. Serum IL-6 level. Bar graphs show cytokine level in pg/ml as mean +/− SEM for 3 samples per group. Data are representative of 3 independent experiments. *, p <0.05; ***, p <0.001.

The optimal maturation of pro-inflammatory DCs leading to the expansion and activation of OVA-specific CD8 T cells and NK cells, were correlated with detectable serum levels of TH1 cytokines. Indeed, twenty hours after the combined CD1d-fusion and OVA/CpG-ODN i.m. injection in mice transferred with Vα14-Jα18 and OT-I cells, IFNγ, TNFα and IL-6 were significantly increased, as compared to each single stimulus (Figure 3). In contrast, the OVA/CpG-ODN alone was able to promote the production of IL-6 and TNFα, indicators of the maturation of pro-inflammatory DCs, but only a small quantity of IFNγ was detected. As expected, the CD1d immunotherapy alone induced a significant production of IFNγ, in addition to TNFα and IL-6, resulting from the concomitant activation of iNKT and NK cells. Noteworthy, at the same time point, the cytokines IL-4 and IL-10 belonging to the TH2/regulatory T cell profile, as well as IL-17a, were undetectable in the sera of all the mice, whatever the treatment. Altogether, the levels of TH1 cytokines detected shortly after the immunization confirmed the efficacy of the combined CD1d-fusion and CpG-based vaccine in activating the innate immune response.

### Enhanced antitumor efficacy of combined CD1d/CpG immunotherapy correlates with accumulation of OVA-specific CTLs at the tumor site

**Figure 4:**
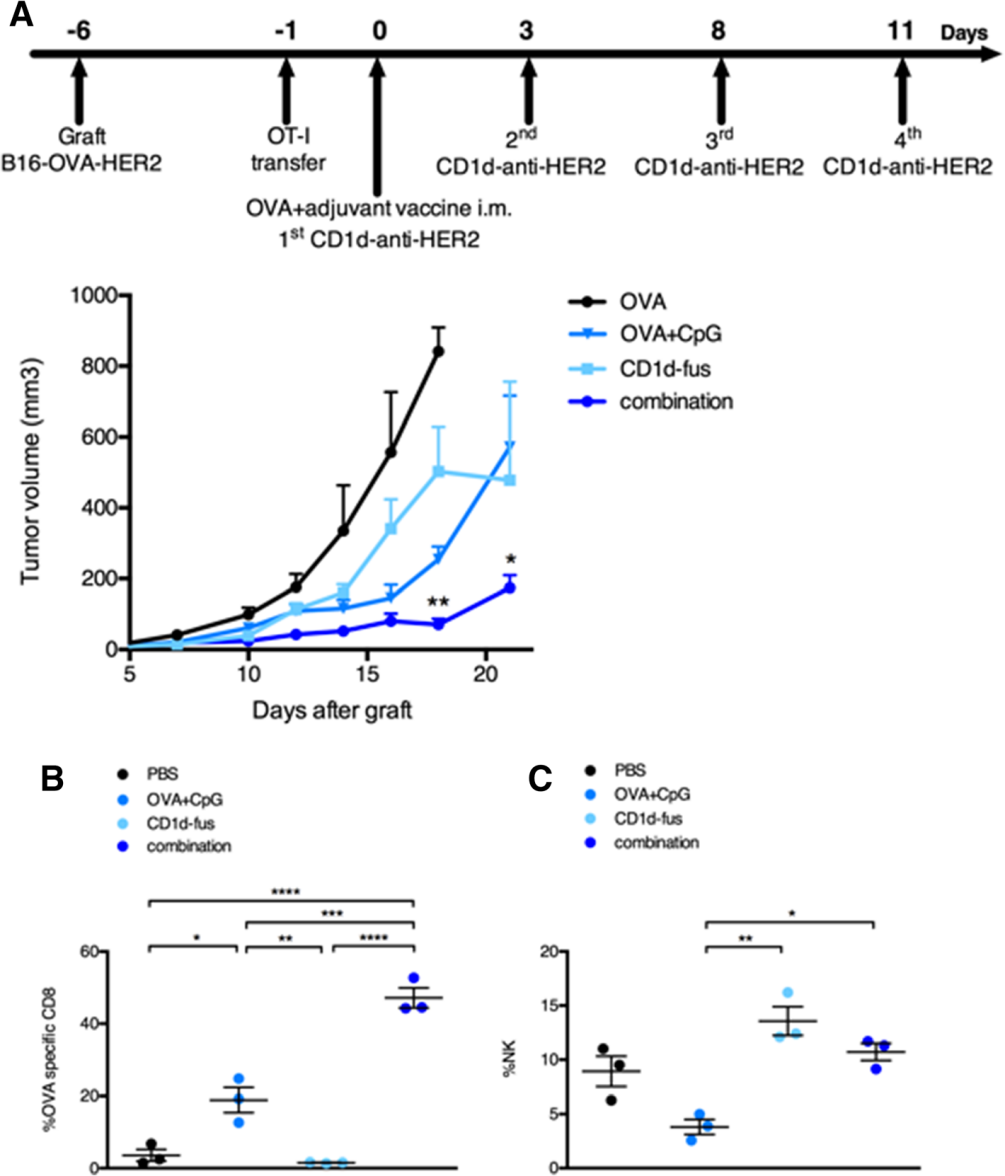
**Combined OVA/CpG vaccine and CD1d-antitumor fusion lead to enhanced antitumor effects correlating with accumulation of OVA-specific CTLs at the tumor site.** Recipient CD45.1 mice were grafted s.c. with B16-OVA-HER2 tumor cells following Vα14-Jα18 and OT-I transfer and 5 days later, they were immunized and treated as described in Figure 1 and in the Methods. **A**. Kinetic of tumor growth shown as mean tumor volume (mm^3^) +/− SEM of 5 mice/group. **B-C**. Mice were sacrificed 7 days after the immunisation and **B**, frequency of H-2K^b^/OVA-specific CD8 T cells among total tumor infiltrating CD8 T lymphocytes and **C**, frequency of NK cells among total tumor lymphocytes were measured. Bar graphs show frequencies as mean +/− SEM of groups of 3 mice. Data are representative of 3 independent experiments. *, p <0.05; **, p <0.01; ***, p <0.001; ****, p <0.0001.

We next assessed the antitumor capacity of the combined CD1d/CpG immunotherapy (Figure 4A). Mice were grafted s.c. with B16-OVA-HER2 tumor cells, that co-express ovalbumin intracellularly and human HER2 on the cell surface following Vα14-Jα18 and OT-I transfer. On day 6, when all tumors were palpable, mice were immunized with the single or combined regimens i.m., and then treated or not with the CD1d-anti-HER2 fusion protein i.v. every three or four days in view of the transient downmodulation of the iNKT TCR [12,17]. As expected from the optimal T cell response, tumor growth was best delayed in mice treated with the combination of CD1d-fusion therapy and OVA/CpG-ODN vaccine (Figure 4A), as compared to the CD1d-fusion therapy or the OVA/CpG-ODN vaccine as single agents. As a negative control, mice immunized with the OVA peptide alone showed fast tumor growth. The importance of OVA-specific CTLs among tumor infiltrating lymphocytes was suggested by a two-fold increase of OVA-specific CD8 T cells when the OVA/CpG-ODN vaccine was combined with the CD1d-therapy compared to the vaccine alone (Figure 4B). Regarding NK cells, the CD1d-fusion therapy induced a significantly increased infiltration of NK cells that was similar whether associated or not with the OVA/CpG-ODN vaccine, suggesting that the superior antitumor activity seen upon the combined treatment, resulted primarily from enhanced CTL-mediated cytotoxicity at this timepoint (Figure 4C).

### Antibody-mediated depletion demonstrates that CD8 T cells rather than NK cells are essential for the combined antitumor effect

**Figure 5:**
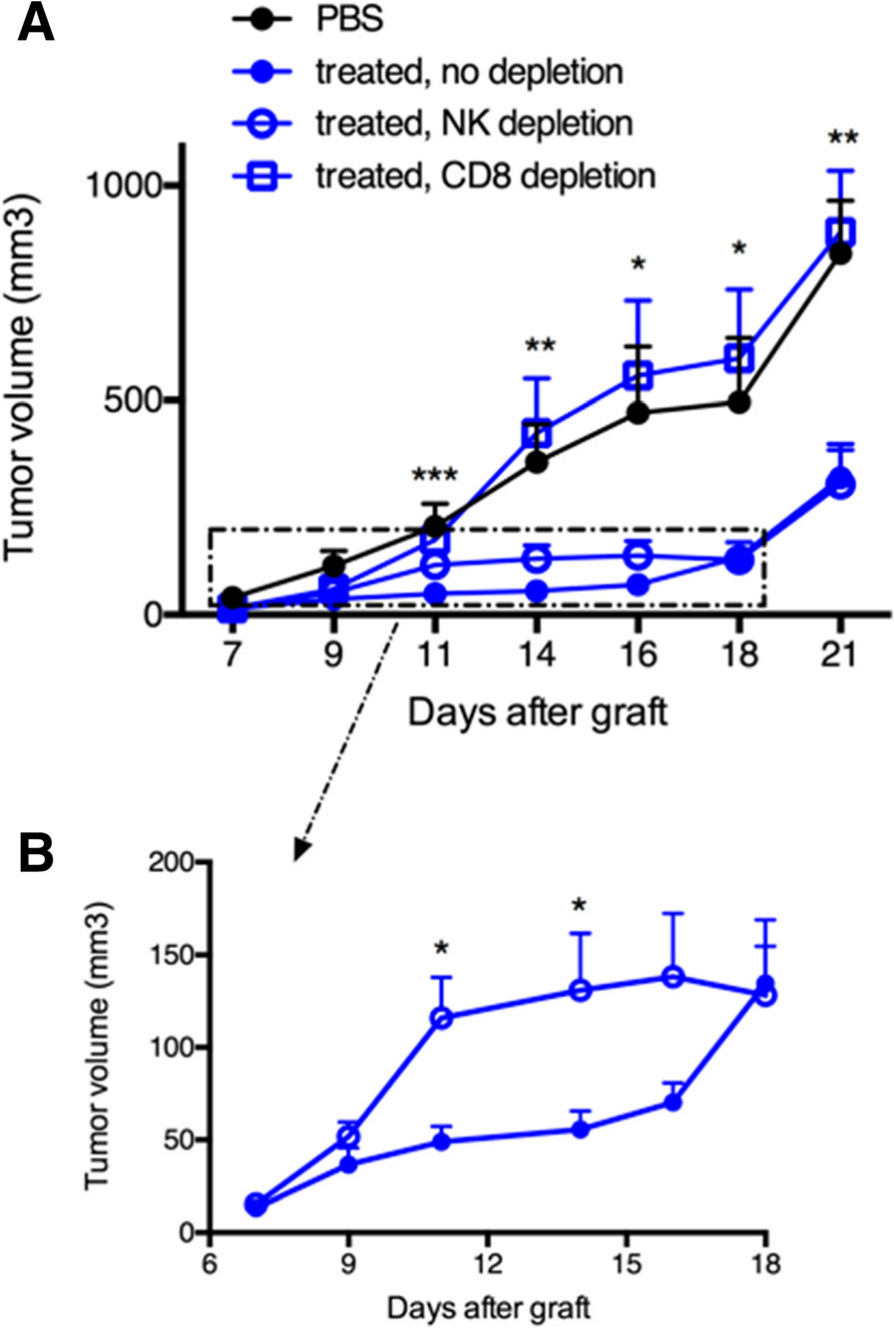
**Antibody-mediated depletion demonstrates that CD8 T cells rather than NK cells are essential for the combined antitumor effect.** Recipient CD45.1 mice were grafted s.c. with 500 000 B16-OVA-HER2 tumor cells following Vα14-Jα18 and OT-I transfer and were immunized and treated 5 days later as described in Figure 1. For depletion of CD8 T cells or NK cells, repeated injections of respectively anti-CD8 or anti-asialo-GM1 antibodies were administered as described in the Methods. **A**. Kinetic of tumor growth shown as mean tumor volume (mm^3^) +/− SEM of 7 mice/group. **B**. Same results zooming in on non-depleted and NK depleted groups. *, p <0.05; **, p <0.01; ***, p <0.001.

To assess the respective role of OVA-specific CTLs and NK cells in the delayed tumor growth, antibody-mediated cell depletion was performed with anti-CD8α or anti-asialo-GM1 antibodies, respectively. Groups of seven mice received Vα14-Jα18 and OT-I cells and one or the other antibody was then administered two days before tumor graft and once every three days thereafter, for a total of four injections. CD1d-antitumor therapy combined with OVA/CpG-ODN vaccination was essentially done as above. Strikingly, the inhibition of tumor growth was completely abolished upon depletion of CD8 T cells, while NK cell depletion had little consequence on the prolonged antitumor effect (Figure 5A). When CD8 T cells were absent a complete loss of antitumor effect was achieved and the tumor growth became similar to that obtained with non-treated mice (Figure 5A). Regarding the role of NK cells, the tumor growth in NK-depleted mice resembled to that of non-depleted mice in the long-term. However, a discrete but visible loss of antitumor activity was observed from day 11 to day 16, implicating a role for NK cells in early tumor regression (Figure 5B). The weak impact of NK cells in the antitumor effects correlated with their low enrichment in the tumor tissues and in parallel, the strong impact of CD8 T cells was associated with a large accumulation of antigen-specific CD8 T cells in tumors. Overall, these data demonstrate the important role of CTLs in the antitumor effects observed for this melanoma model after the combined CD1d-immunotherapy and OVA/CpG-ODN vaccine.

## Discussion

In most cases, immature DCs will induce tolerance when encountering a tumor-derived self-antigen, unless danger signals are provided. Therefore, one key element for a successful vaccine formulation is to favor the maturation of pro-inflammatory DCs in order to induce a potent adaptive immune response against cancer cells. To this aim, TLR ligands represent potent danger signals able to induce the maturation of TH1-biased DCs, and are often involved in cancer vaccine formulations [18]. We recently demonstrated that peptide antigens combined in particular with Poly(I:C) (TLR-3) or CpG ODN (TLR-9) were optimal formulations leading to higher IL-12 serum levels and increased Teff to Treg ratio, which in turn resulted in increased antitumor effects, as compared to other TLR ligands [4]. However, despite the success of cancer vaccines in mouse models, therapeutic vaccination strategies have had only limited success in humans. In 2004, Rosenberg et al. analysed the outcome of 86 therapeutic vaccination trials in a range of different cancers, and discovered an overall objective clinical response rate of only 3.3% [19]. A more recent review showed some promising advances with therapeutic vaccines but complete clinical responses are still rare [20]. The most successful vaccination strategies appear to be those where vaccines are combined with chemotherapy, immune check-point blockade or novel adjuvant formulations, indicating that combination therapies are the way of future vaccine design.

In the present study, the antitumor therapeutic effects of peptide/CpG ODN vaccine were further enhanced by combining with the specific activation of iNKT cells via αGC/sCD1d-antitumor fusion proteins, which we previously developed to redirect the innate and adaptive immune responses to the tumor site [11,12]. Most strikingly, the combined treatment resulted in a synergistic increase in serum IL-12, denoting an optimal maturation of pro-inflammatory DCs. The second major observation was that CD8α^+^ DC appeared to be the major responders to the combined immunization with peptide/CpG/CD1d-fusion, as seen especially by their much superior increase in CD40 and to a lesser extent in CD86 expression, when compared to CD8α^neg^ and pDC subsets. Recent studies have already demonstrated the tight collaboration between iNKT cells and cross-presenting CD8α^+^ DCs, and their importance in generating long-term CD8 T cell memory [21,22]. The immunoregulatory role of CD1d-restricted iNKT cells in the transactivation of innate and adaptive immune responses has been well demonstrated [7,23], and several studies have exploited iNKT cells for therapeutic cancer vaccination [24,25]. One major advantage is that iNKT cells represent a readily available source of help for DCs, as they do not require antigen priming like naïve CD4 T cells do [26]. The crosstalk between iNKT cells and DCs depends on both the invariant TCR-CD1d/glycolipid antigen and CD40L-CD40 interaction, which will guarantee a subsequent efficient T cell priming [27]. Indeed, we show in the present study that upon combined peptide/CpG-ODN vaccine and αGC/CD1d-antitumor fusion protein, DC maturation markers including CD40, CD86 and CD70 were significantly increased as compared to individual treatment. In addition to DC maturation, the combined peptide/CpG-ODN vaccine and αGC/sCD1d-antitumor fusion protein also resulted in a synergistic expansion of NK cells as seen in the spleen on day 7. iNKT cells are well-known to quickly transactivate NK cells directly via their release of IFNγ and indirectly via their crosstalk with DCs [28,29]. In this context, the strong IL-12 release by DCs upon the combined treatment in addition to IFNγ, likely explains the large expansion of NK cells. Importantly, in view of their respective adjuvant effect on the adaptive immune response, we also observed a cooperative effect of peptide/CpG-ODN and αGC/CD1d-antitumor treatment on the expansion of H-2Kb/OVA specific CD8 T cells. Altogether, the enhanced innate and adaptive responses to the combined treatment correlated well with significantly higher serum levels of TH1 cytokines such as IFNγ, TNFα and IL-6. This cooperative effect was also observed when triggering the expansion of endogenous H-2Kb/Trp2 specific CD8 T cells, as described in Additional file 3: Figure S3. However, while the large expansion of NK cells was just as robust as that observed in the OT-I/Vα14-Jα18 transfer model, the frequencies of Trp-2-specific T cells remained about fivefold less than for OT-I T cells. This weaker frequency possibly reflects the more stringent conditions required to induce the expansion of tumor/self antigen-specific T cells, which are likely to be present at a low frequency and to have a high activation threshold compared to transgenic CD8^+^ T cells. This highlights the advantage of combining a CD8 T cell vaccine with CD1d-fusion protein, which harnesses the innate immune response to boost the adaptive response and enhance anti-tumor immunity. These combined adjuvant effects between TLR ligands and the CD1d superagonist αGC have been reported previously for TLR-3, 4 and 9 [9,30]. However, the use in the present study of αGC-loaded CD1d-antitumor fusion proteins, instead of αGC as a free drug, offers two promising advantages. Firstly, combining the CD1d-antitumor therapy with tumor vaccines allows the prolonged reactivity of iNKT cells to multiple injections of αGC-loaded CD1d fusion proteins thus sustaining the anti-tumor immune response [12]. This is in contrast to the iNKT cell anergy that can already be induced by a single injection of free αGC, as shown in a large number of pre-clinical studies [12,17,31–33]. Clinical trials in cancer patients have preferred the autologous transfer of αGC-pulsed DC, which showed prolonged iNKT cell activation, as compared to the glycolipid alone [34–38]. However, clinical responses remained very limited unless the cancer was directly targeted by infusion of the cells in the proximity of the tumor [38,39]. In these trials, clinical responses correlated with the frequency of tumor infiltrating T cells, indicating the importance of tumor targeted immunotherapy. Thus, the tumor targeting of αGC is another important advantage of αGC/sCD1d-antitumor fusion proteins as combinatorial vaccine agents. We have previously demonstrated that these tumor targeted fusion proteins are able to induce direct NKT cell killing of tumor cell lines bearing the specific target antigen and to redirect both the innate and adaptive responses to the tumor site, which greatly improves antitumor efficacy [11,12]. Consistent with our previous work, the present results confirmed the accumulation of both innate and adaptive effector subsets in the tumor tissue when targeted with the combined vaccine and CD1d-anti-HER2 fusion protein therapy. NK cells increased in the tumor with or without the OVA/CpG-ODN vaccine, but most importantly OVA-specific CTL numbers increased two-fold in the tumors upon combined OVA/CpG-ODN vaccine and CD1d-anti-HER2 therapy as compared to vaccine alone. The increase in CTL was greater than the sum of the individual responses of the vaccine and the CD1d-anti-HER2. This indicates a synergy between the two treatments, which correlates with the improved inhibition of tumor growth. Importantly, the therapeutic efficacy of the combined therapy was completely abolished upon CD8 T cell depletion but not NK cell depletion, suggesting a strict requirement of CD8 T cells. Unexpectedly, these results were in contrast with previous reports from us and others, demonstrating that iNKT-cell mediated antitumor activity was dependent on the transactivation of NK cells [12]. However, when zooming in on the kinetic of tumor growth with or without NK cell depletion, their involvement in tumor inhibition was clearly visible until day 18, when tumor escape usually occurs in these tumor settings [12,31,40]. The addition of OVA/CpG-ODN immunization induced the activation and potent expansion of the adoptively transferred OVA-specific CTLs thus allowing tumor escape to be further delayed.

## Conclusions

In conclusion, combined vaccine/CD1d immunotherapy enhances the activation and recruitment of tumor-specific CD8 T cells, which appears to be favored by the local inflammation induced by activated iNKT and NK cell accumulation at the tumor site. Thus, the optimized DC maturation and antigen-presentation at the tumor site upon repeated CD1d-mediated immunotherapy has the potential to prolong the anti-tumor CTL response, in addition to providing an additional adjuvant boost to the initial active antitumor vaccination. It is becoming evident that the combination of different therapies is the most promising tool available to improve the clinical outcome of existing therapeutic cancer vaccines, and here we demonstrate a way to enhance vaccine efficacy by harnessing two anti-tumor immune pathways to produce a powerful synergistic anti-tumor effect.

## Methods

### Animals, cell lines, vaccine and other reagents

Mice were maintained at the University of Lausanne’s Specific Pathogen Free Unit. C57BL/6 mice were obtained from Harlan Laboratories (The Netherlands), and CD45.1 congenic B6.SJL-PtprcaPep3b/BoyJArc breeders were purchased from Charles River, L’Arbresle (France), and bred on site. TCR-transgenic OT-I mice were originally obtained from Jackson Laboratories (USA), and bred on site. Vα14-Jα18 TCR transgenic mice were kindly provided by A. Bendelac, University of Chicago (USA) [41], and were bred on site. Age and sex-matched mice between 6–14 weeks of age were used for all experiments. This study was approved by the Veterinary authority of the Canton of Vaud (Permit #1605), and experiments were performed in accordance with Swiss ethical guidelines.

The B16 melanoma cell line was stably transduced with the retroviral vector pLPcx containing the human HER2, and subsequently transfected with pcDNA3.1 containing the cytoplasmic part of ovalbumin (residues 45–378 [42]). The double positive B16-OVA/HER2 cell line was maintained under puromycin and neomycin selection. The H-2K^b^-restricted OVA_257–264_ epitope was synthetized at the Protein and Peptide Chemistry Facility of the University of Lausanne. CpG-ODN 1826 (Class B) was purchased from InvivoGen Europe (France). The CD1d super agonist α-galactosylceramide (αGC) KRN7000 was purchased from Enzo Life Science.

### Recombinant CD1d-anti-HER2 fusion protein and CD1d tetramer

The CD1d-anti-HER2 fusion protein was essentially produced and purified as described previously [12]. Shortly before systemic treatment, proteins were loaded with a 2.5 fold excess of αGC KRN7000, and unbound glycolipid was removed by size exclusion FPLC. Integrity and absence of aggregates, as well as endotoxin contamination were regularly checked by respectively SDS-PAGE, FPLC and LAL bioassay. Recombinant proteins were used if endotoxin level was <0.5EU/μg protein corresponding to <0.04 ng endotoxin/μg of protein. The CD1d tetramer was produced as previously described [11].

### Flow cytometry analysis and cytokine bead array analysis

Cells were stained first with the MHC-class-I/SIINFEKL tetramer at RT for 30 min, then, CD1d tetramer was added for additional 30 min on ice. Surface staining antibodies were added without wash and incubated for 20 min on ice. Cells were washed once with PBS 2%FCS and resuspended in 200 μl of PBS 2%FCS for acquisition. All fluorochrome-labeled antibodies were purchased from Becton Dickinson (BD Biosciences) or eBiosciences. Flow cytometric analyses were performed with a FACSCalibur, FACSCanto or LSRII cytometer (BD Biosciences) and the acquired data were processed using FlowJo software (Tree Star Inc.). Cytokine levels were measured by fluorescence-based multiplex assay using BD Cytometric Bead Array kit TH1/TH2/TH17 or BD Mouse IL-12p70 Flex Set Kit (CBA, BD Biosciences).

### Adoptive cell transfer, immunisations and treatments

OVA-specific CD8 T cells and iNKT cells (CD45.2) were isolated from spleens of OT-I and Vα14-Jα18 mice, respectively. Single cell suspensions were obtained by disrupting the lymphoid tissue and the frequency of transgenic T cells was determined by flow cytometry. Cells were labeled with Vα2 and β5.1/5.2 antibodies for OT-I cells and CD1d tetramers and CD3 antibody for NKT cells. Naive B6-SJ ptp-rca (CD45.1) recipient mice received an i.v. transfer of 1x10^5^ OT-I cells and 5 × 10^5^ NKT in 200 μl DMEM. One day after the transfer, mice were vaccinated intra-muscularly (i.m.) with 50 μl of either PBS, OVA peptide alone (10 μg), OVA peptide (10 μg) plus CpG-ODN (50 μg) alone, or in combination with αGC/CD1d-scFv fusion protein (40 μg). Vaccination was immediately followed by i.v. treatments with 200 μl of either PBS alone or αGC/CD1d-antitumor scFv fusion proteins (40 μg). Systemic treatment with the αGC/CD1d-antitumor scFv fusion proteins (40 μg) was repeated at 2 to 4-day intervals. This schedule was optimized based on the NKT TCR recycling kinetics following activation/stimulation as previously published [12].

### DC maturation

Spleens were harvested 20 hours after the immunisations and disrupted to obtain a single-cell suspension. Splenocytes were next enriched for CD11c^+^ cells using the autoMACS system (Miltenyi Biotec, Germany). Briefly, cells were labeled with anti-CD11c microbeads (Miltenyi Biotec), as per the manufacturer’s protocol, and purified using the POSSEL program on the AutoMACS. The positive fraction was recovered for maturation marker analysis by flow cytometry.

### Tumor immunotherapy

CD45.1 congenic mice were grafted s.c. in the right flank with 5 × 10^5^ B16-OVA-HER2 cells. Five days after the graft, a mix of 5 × 10^5^ Vα14-Jα18 TCR-transgenic.

NKT cells and 1 × 10^5^ OT-I CD8 T cells were transferred into the mice. Immunisations were performed six days after the graft as described above. Mean tumor volume, monitored every two days, was calculated using the following formula (length × width × thickness)/2.

### Tumor-infiltrating lymphocyte analysis

At day 7 after the vaccination, tumors were harvested and digested for 40 min at 37°C, according to the Tumor dissociation kit protocol (MACS Miltenyi Biotec, Germany). Tumors were crushed on 100 μm cell strainers and washed twice with PBS 2%FCS. Single cell suspensions were enriched for CD45+ cells using the autoMACS system (Miltenyi Biotec, Germany). Briefly, cells from maximum 500 mg of tumor tissue were labeled with anti-CD45 microbeads (Miltenyi Biotec), as per the manufacturer’s protocol, and purified using the POSSEL program on the AutoMACS. The positive fraction was recovered for TILs analysis by flow cytometry.

### Antibody-mediated CD8 and NK cell depletion

CD8 T cells were depleted by repeated i.p. injections of 100 μg of anti-mouse CD8α mAb, clone 53–6.72 (BioXCell, USA). NK cells were depleted by repeated injections of 50 μl of mouse anti-asialo-GM1 polyclonal Abs suspension (Wako Pure Chemical). Anti-asialo-GM1 antibodies were used as an alternative to anti-NK1.1, to avoid the concomitant depletion of a large fraction of iNKT cells, which are NK1.1^+^, while ensuring specific depletion of NK cells. The first injection was done two days before the immunisations and repeated every three or five days for a total of four injections.

### Statistical analysis

Results are expressed as mean ± SEM. Statistical significance was determined with the one-way-ANOVA test with Bonferroni correction (GraphPad Prism, GraphPad software). Tumor progression statistics were calculated with the unpaired *t*-test for each time-point (GraphPad Prism, GraphPad software). (*, p <0.05; **, p <0.01; ***, p <0.001; ****, p <0.0001).

## Additional files

Additional file 1: Figure S1. Description of the gating strategy to discriminate CD8α^+^ and CD8α^neg^ DCs as well as pDCs is shown. Live splenocytes from experiment described in Figure 1 were stained for B220, CD11c, CD11b, MHC II and CD8α. Conventional DCs were gated as B220^neg^CD11c^high^ and CD8α^+^ or CD8α^neg^. pDCs were identified as B220^+^CD11c^+^. Additional file 2: Figure S2. The αGC/CD1d-anti-HER2 fusion cooperates with CpG-ODN to rapidly induce iNKT-mediated activation of NK cells. A. Mice transferred with Vα14-Jα18 and OT-I cells were immunized i.m. with OVA peptide alone or in combination with CpG or CD1d-fus/CpG followed or not by systemic treatment with the CD1d-antitumor protein. The *ex vivo* expression of CD69 on NK cells was determined twenty hours post indicated treatments. Bar graph represents mean of fluorescence of CD69 on the CD3negNK1.1+ cell population. B. 2 × 10^5^ splenocytes from a Vα14-Jα18 transgenic mouse were cultured in complete DMEM medium in presence of either plate-coated αGC/CD1d-anti-HER2 fusion (40 μg/ml), CpG-ODN (5 μg/ml) or the combination of the two stimuli. Cells were recovered at 6hours and analyzed by flow cytometry. Bar graph represents mean of fluorescence of CD69 on CD1d tetramer+ CD3+ iNKT cell population. ***, p < 0.001; ****, p < 0.0001. Additional file 3: Figure S3. Cooperative effects of Trp2 peptide/CpG ODN and CD1d-anti-HER2 fusion protein independently of T and iNKT adoptive cell transfer. Naive C57BL/6 mice were vaccinated with 20 μg of a short immunogenic peptide from Tyrosinase-related protein 2 (Trp2 180-188) alone, with CpG-ODN (50 μg) or with a combination of CpG and CD1d-anti-HER2 fusion (40 μg). Mice were bled 7 days after the immunization. A. Frequency of H-2Kb/Trp2-specific T cells among circulating CD8^+^ lymphocytes. B. Frequency of NK cells among total lymphocytes in the blood. Scatter dot plot graphs show frequencies as mean +/- SEM of groups of 8 mice. Data are representative of two independent experiments. *, p < 0.05; ***, p < 0.001.
