# Supplementary figures and images for: iNKT/CD1d-antitumor immunotherapy significantly increases the efficacy of therapeutic CpG/peptide-based cancer vaccine
Source: J Immunother Cancer. 2014 Nov 18;2:39. doi: 10.1186/s40425-014-0039-8 (PMC4243737; doi:10.1186/s40425-014-0039-8)

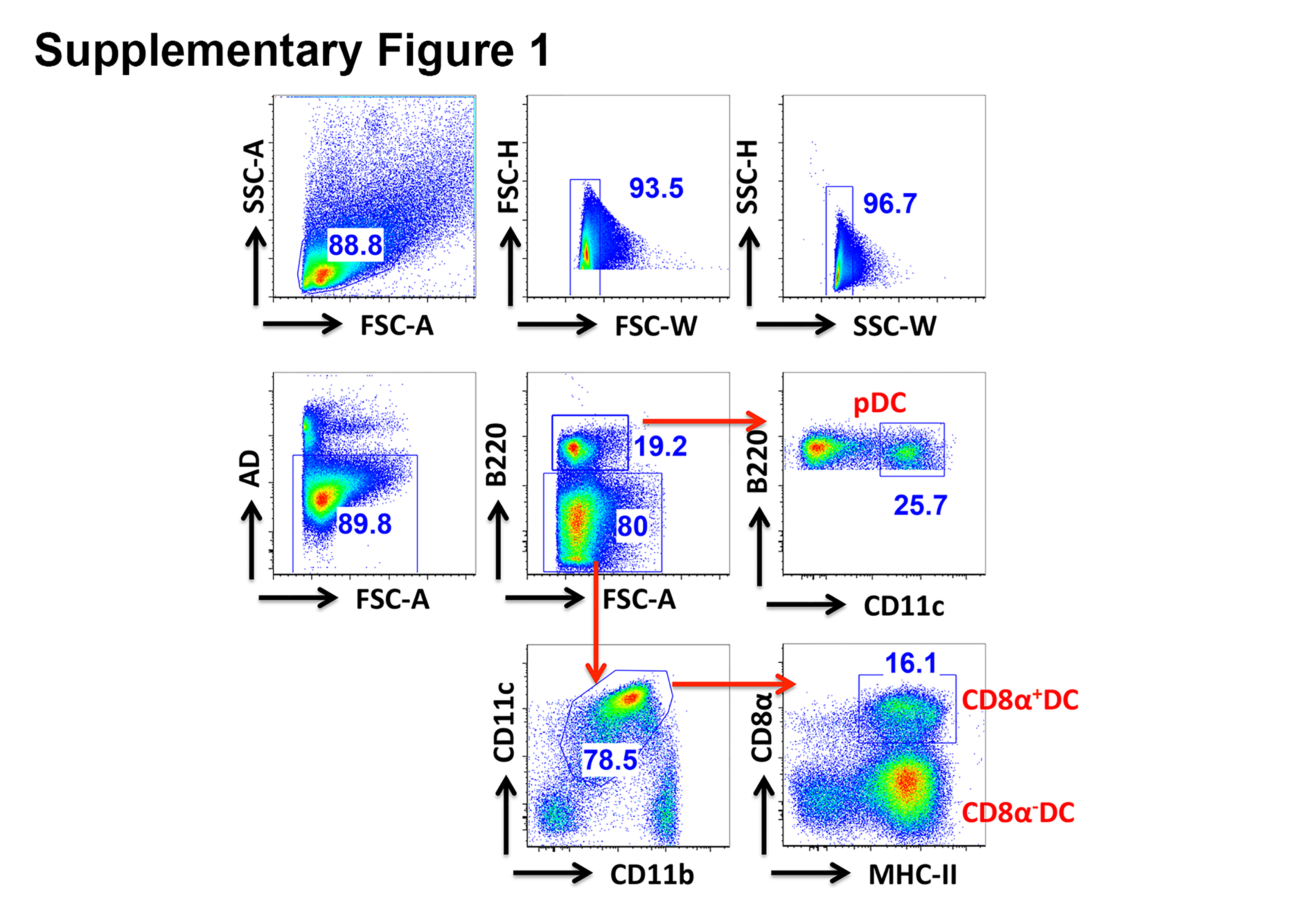

Supplement: Additional file 1: Figure S1. — Description of the gating strategy to discriminate CD8α+ and CD8αneg DCs as well as pDCs is shown. Live splenocytes from experiment described in Figure 1 were stained for B220, CD11c, CD11b, MHC II and CD8α. Conventional DCs were gated as B220negCD11chigh and CD8α+ or CD8αneg. pDCs were identified as B220+CD11c+. [file 40425_2014_39_MOESM1_ESM.tif]

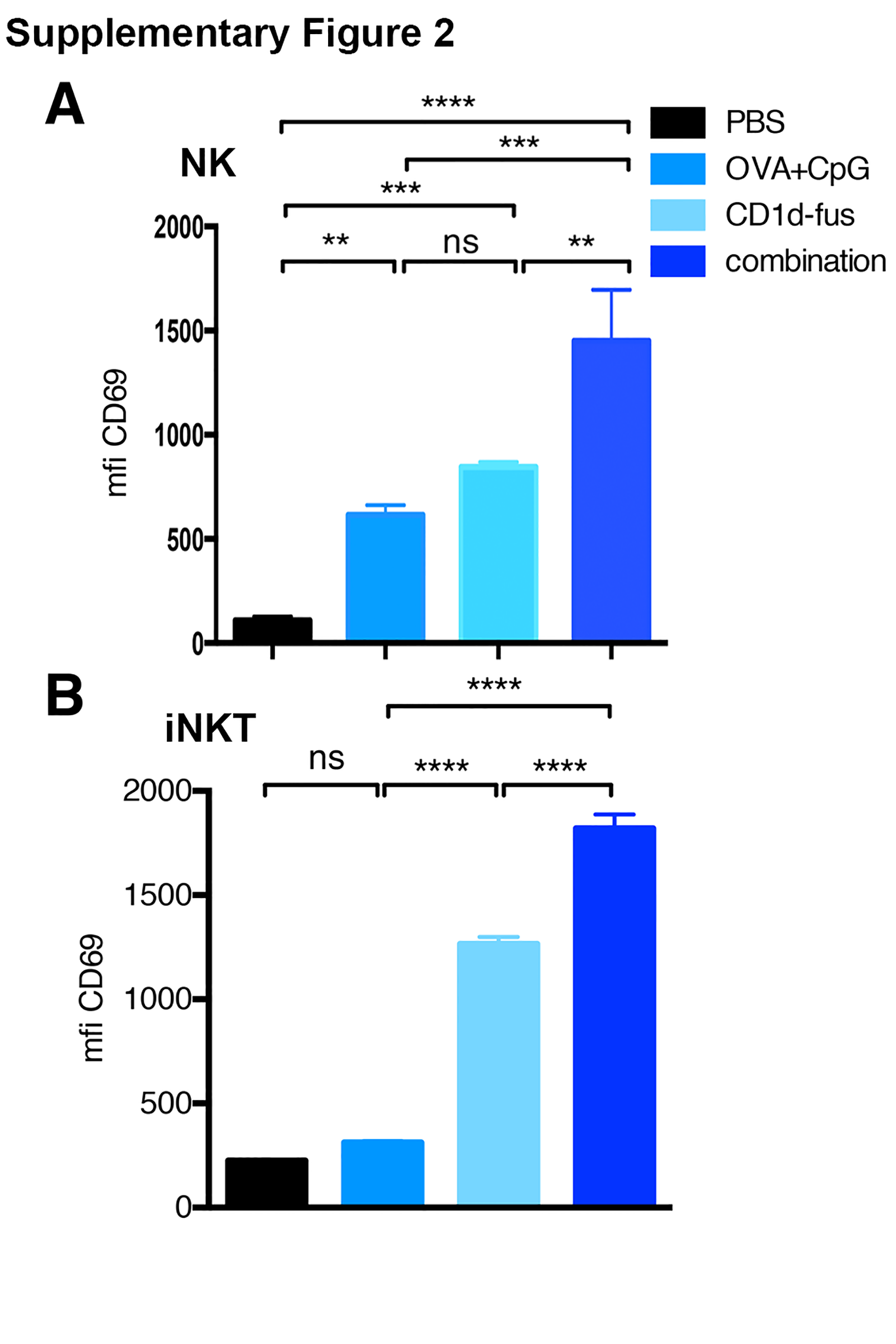

Supplement: Additional file 2: Figure S2. — The αGC/CD1d-anti-HER2 fusion cooperates with CpG-ODN to rapidly induce iNKT-mediated activation of NK cells. A. Mice transferred with Vα14-Jα18 and OT-I cells were immunized i.m. with OVA peptide alone or in combination with CpG or CD1d-fus/CpG followed or not by systemic treatment with the CD1d-antitumor protein. The ex vivo expression of CD69 on NK cells was determined twenty hours post indicated treatments. Bar graph represents mean of fluorescence of CD69 on the CD3negNK1.1+ cell population. B. 2 × 10^5^ splenocytes from a Vα14-Jα18 transgenic mouse were cultured in complete DMEM medium in presence of either plate-coated αGC/CD1d-anti-HER2 fusion (40 μg/ml), CpG-ODN (5 μg/ml) or the combination of the two stimuli. Cells were recovered at 6hours and analyzed by flow cytometry. Bar graph represents mean of fluorescence of CD69 on CD1d tetramer+ CD3+ iNKT cell population. ***, p < 0.001; ****, p < 0.0001. [file 40425_2014_39_MOESM2_ESM.tif]

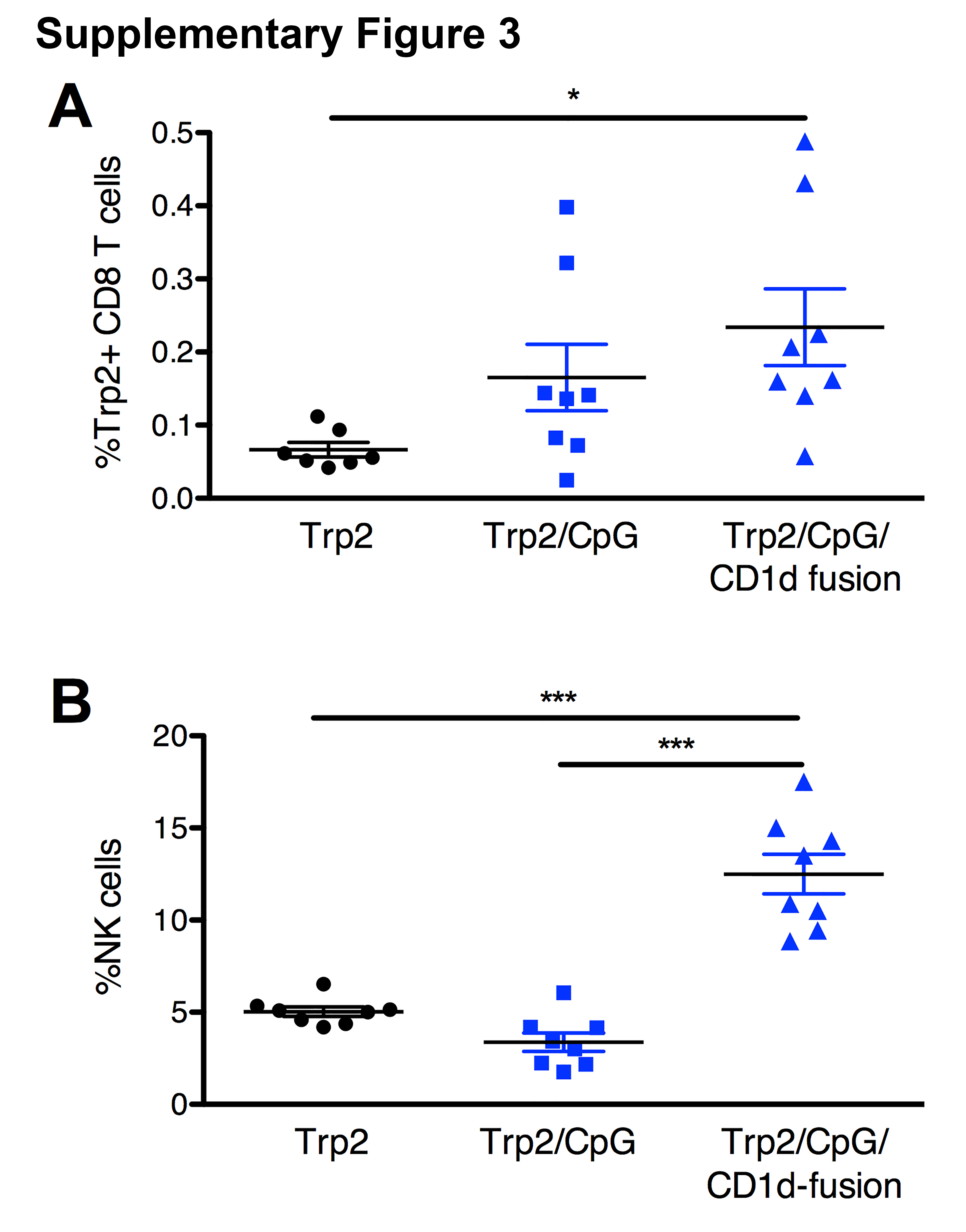

Supplement: Additional file 3: Figure S3. — Cooperative effects of Trp2 peptide/CpG ODN and CD1d-anti-HER2 fusion protein independently of T and iNKT adoptive cell transfer. Naive C57BL/6 mice were vaccinated with 20 μg of a short immunogenic peptide from Tyrosinase-related protein 2 (Trp2 180-188) alone, with CpG-ODN (50 μg) or with a combination of CpG and CD1d-anti-HER2 fusion (40 μg). Mice were bled 7 days after the immunization. A. Frequency of H-2Kb/Trp2-specific T cells among circulating CD8+ lymphocytes. B. Frequency of NK cells among total lymphocytes in the blood. Scatter dot plot graphs show frequencies as mean +/- SEM of groups of 8 mice. Data are representative of two independent experiments. *, p < 0.05; ***, p < 0.001. [file 40425_2014_39_MOESM3_ESM.tif]
